# Sonographic Crepitus, a Point-of-Care Ultrasound Finding

**DOI:** 10.24908/pocus.v7i1.15406

**Published:** 2022-04-21

**Authors:** Brian Kohen, Michael Halperin, Gloria Felix, Trevor Dixon, Michelle Montenegro, Fenil Patel

**Affiliations:** 1 Albert Einstein College of Medicine, Jacobi Medical Center

**Keywords:** Ultrasound, POCUS, Crepitus, Necrotizing Fasciitis, Point-of-care, Emergency Medicine

## Introduction

Necrotizing fasciitis is a life-threatening polymicrobial skin and soft tissue infection that requires prompt diagnosis and treatment. Delays in diagnosis and treatment can result in an increase in morbidity and mortality 1. Necrotizing fasciitis has historically been a clinical diagnosis. Patients with a high clinical suspicion for necrotizing fasciitis generally receive antibiotics and undergo emergent surgical debridement. In some cases, necrotizing fasciitis may be clinically difficult to differentiate from other skin and soft tissue infections such as severe cellulitis and abscesses. In such cases, POCUS may assist in diagnosis and has been shown to have a positive impact in expediting care 2, 3. Below, we describe a unique sonographic finding in a patient diagnosed with necrotizing fasciitis. 

## Presentation and Discussion

**Figure 1:**
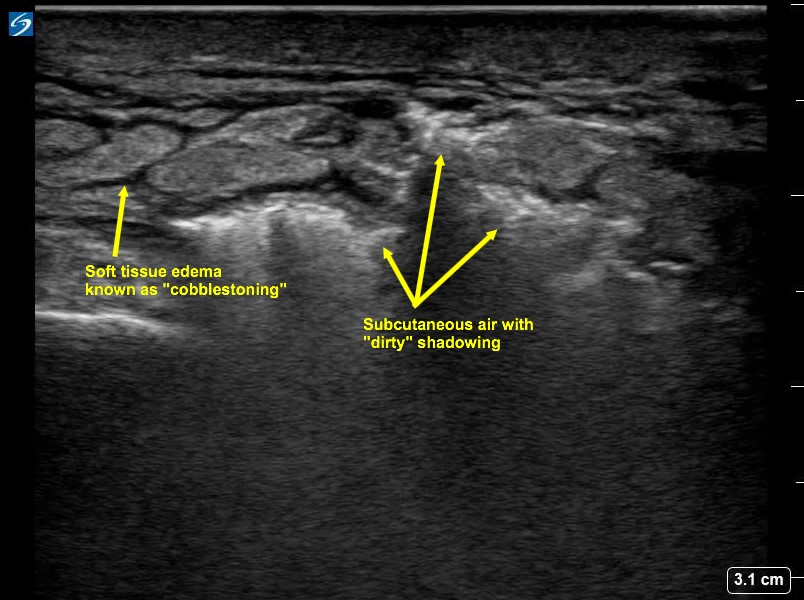
Cobblestoning and hyperechoic subcutaneous air with “dirty” shadowing, suggestive of necrotizing fasciitis.

A 62-year-old male with no reported past medical history presented with worsening left foot swelling after cutting his toenail. Abnormal vital signs included a blood pressure of 166/99 mmHg, heart rate of 121 beats per minute, and a fingerstick blood glucose of 500 mg/dL. On physical examination, his left foot was swollen, warm, erythematous, and tender to palpation. There was a poorly healing wound on the plantar surface of his left foot. Of note, crepitus was not felt. A point-of-care ultrasound (POCUS) of the left foot was performed which showed extensive cobblestoning without a discrete fluid collection, and deeper “dirty” shadowing suggestive of subcutaneous air (Figure 1). When gentle pressure was applied with the transducer, the subcutaneous air mobilized, confirming our suspicion that the “dirty” shadowing visualized was indeed subcutaneous air (Video S1). We call this novel sonographic finding “sonographic crepitus.” This dynamic visualization of subcutaneous air movement with transducer pressure application ultimately raised our suspicion for necrotizing fasciitis, in an otherwise equivocal physical examination of the wound. Prior studies have described sonographic findings consistent with necrotizing fasciitis such as subcutaneous thickening, air, and fascial fluid as well as an approach to early POCUS screening in these patients 2, 3. We hope that sonographic crepitus may be added to the continuum of sonographic findings associated with necrotizing fasciitis and further assist diagnosis in ambiguous cases.

## Clinical follow up

**Figure 2:**
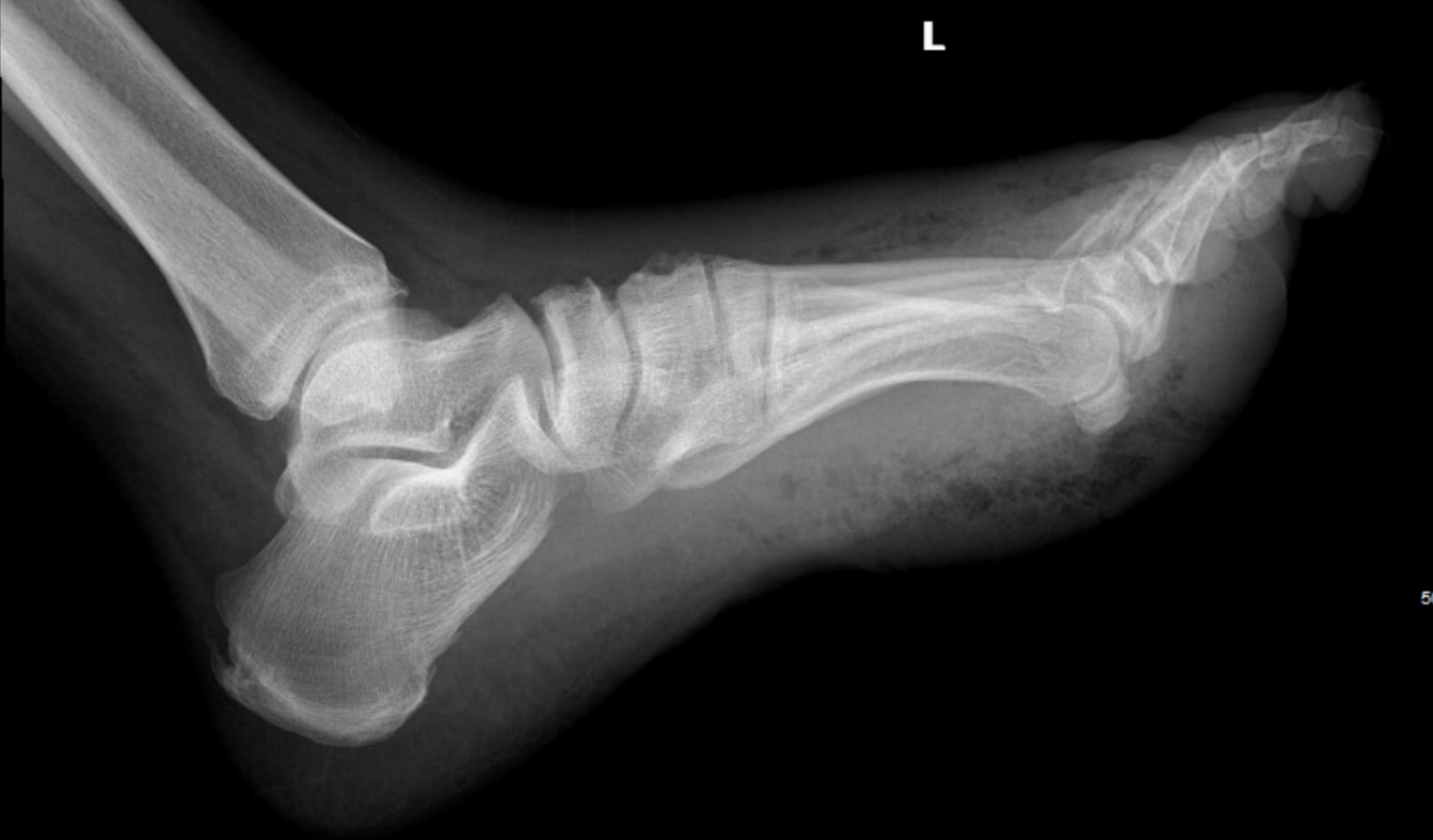
Lateral view of a plain radiograph showing extensive subcutaneous air concerning for necrotizing fasciitis.

In the emergency department, an X-ray of the patient’s foot was performed revealing diffuse soft tissue gas (Figure 2). From the emergency department, he was taken to the operating room for a transmetatarsal amputation. A surgical wound culture grew multiple organisms including Enterococcus faecalis, Bacteroides fragilis, Streptococcus mitis, Streptococcus oralis, and Streptococcus constellatus. Four days later, he was discharged to an acute rehabilitation facility with a wound vacuum. One month after the initial presentation to the emergency department, he was discharged to home care. He was recently discharged from home care services and currently follows with general surgery and wound care specialists as an outpatient, with improvement in his wound healing.

## Limitations

The POCUS for this patient was performed by an emergency ultrasound-trained physician, who was able to identify the abnormal sonographic findings seen in necrotizing fasciitis. The ability to properly operate POCUS and identify these findings requires additional training that may not be ubiquitous.

## Future

We hope this sonographic finding sparks interest to obtain additional data on sensitivities, specificities, positive predictive value, and negative predictive value of sonographic crepitus.

## Conclusion

Sonographic crepitus, or mobilization of dirty air shadowing with application of probe pressure to the affected area, is a sonographic finding we describe here, in a patient diagnosed with necrotizing fasciitis. Questions still remain regarding the clinical utility and efficacy of POCUS in the diagnosis of patients with necrotizing fasciitis, an historically clinical diagnosis. We emphasize that obtaining a POCUS should not delay definitive treatment, though, in equivocal cases, it can serve as an additional diagnostic tool. 

## Disclosures

None.

## Supplementary Material

 Video S1 Real-time visualization of mobilizing hyperechoic subcutaneous air with transducer pressure.
